# Individual luteolysis pattern after GnRH-agonist trigger for final oocyte maturation

**DOI:** 10.1371/journal.pone.0176600

**Published:** 2017-05-01

**Authors:** Barbara Lawrenz, Nicolas Garrido, Suzan Samir, Francisco Ruiz, Laura Melado, Human M. Fatemi

**Affiliations:** 1 IVF department, IVI Middle-East Fertility Clinic, Abu Dhabi, UAE; 2 Obstetrical Department, Women´s university hospital Tuebingen, Tuebingen, Germany; 3 Statistical Department, IVI Foundation, Valencia, Spain; University of Crete, GREECE

## Abstract

Final oocyte maturation using GnRH-agonist trigger in a GnRH-antagonist protocol is increasingly common, as ovarian hyperstimulation syndrome is almost completely avoided. However, this approach might lead to reduced pregnancy rates due to severe luteolysis. This proof of concept study evaluated the extend of luteolysis by measuring progesterone levels 48 hours after oocyte retrieval in 51 patients, who received GnRH-agonist trigger for final oocyte maturation in a GnRH-antagonist protocol due to the risk of ovarian hyperstimulation syndrome. It was shown, that luteolysis after GnRHa-trigger differs greatly among patients, with progesterone levels ranging from 13.0 ng/ml to ≥ 60.0 ng/ml, 48 hours after oocyte retrieval. Significant positive correlations could be demonstrated between progesterone levels and the number of ovarian stimulation and suppression days (p = 0.006 and p = 0.002 respectively), the total amount of medication used for ovarian suppression (p = 0.015), the level of progesterone on the day of final oocyte maturation (p = 0.008) and the number of retrieved oocytes (p = 0.019). Therefore it was concluded, that luteolysis after GnRH-agonist trigger is patient-specific and also luteal phase support requires individualization. Longer stimulation duration as well as a higher level of progesterone on the day of final oocyte maturation and more retrieved oocytes will result in higher levels of progesterone 48 hours after oocyte retrieval.

## Introduction

In stimulated IVF cycles, final oocyte maturation is a crucial step to plan retrieving oocytes from preovulatory follicles.

HCG (human Chorion Gonadotropin) is usually administered for final oocyte maturation to mimic the midcycle LH (Luteinising Hormone) surge, however, in recent years, the use of GnRH (Gonadotropin-releasing-hormone)-agonist in GnRH-antagonist protocols has become increasingly common. Advantages of this approach are the significant reduction of ovarian hyperstimulation syndrome (OHSS) [1] and the possible increase of the number of mature oocytes [2].

HCG and LH activate the same receptor and are therefore capable to induce final oocyte maturation. The main important difference between LH and hCG is the significant difference in half-life, which is <60 minutes for LH and >24 hours for hCG [3,4]. This is especially important regarding the potential to induce OHSS.

In contrast to the application of hCG, which imitates the mid-cycle LH-surge, administration of GnRH-agonist dislocates the GnRH antagonist from the GnRH receptors in the pituitary, leading to a surge of LH and FSH and therefore mimicking the surge of gonadotropins in the natural cycle [5]. Whether FSH has a physiologic role in oocyte maturation is not known, however, it was suggested that the addition of FSH might improve oocyte recovery and fertilization rates [6]. The LH-surge triggered through GnRH-agonist is effective to induce final oocyte maturation and [7–9] and is considered to be more physiologic compared to final oocyte maturation with hCG [10].

Despite the fact that the application of a GnRH-agonist-trigger in a GnRH-antagonist-protocol imitates the natural mid-cycle surge of LH and FSH, distinct differences in the LH-surges are found. The spontaneous LH-surge in a natural cycle is characterized by an ascending phase of approximately 4 hours, a peak plateau of 20 hours, and a descending phase of 20 hours whereas the GnRHa-induced LH/FSH surge has a significantly shorter ascending phase.

The magnitude of both LH-surges are comparable [8]. The differences between natural and artificial induced LH-surge cannot be overcome by an increase in the GnRH-agonist dosage or by repeat GnRH-agonist-administration [11].

Besides inducing final oocyte maturation, a bolus of GnRH-agonist also acts as a luteolytic agent and prevents the secretion of vasoactive substances, mainly VEGF, from the corpora lutea, which is considered an important target factor in order to avoid OHSS [12]. The GnRH-trigger for final oocyte maturation causes a direct pituitary down-regulation and therefore, the remaining corpora lutea lack LH-stimulus [13] and are not capable to sustain progesterone production for the luteal phase. Additionally, LH secretion from the pituitary is suppressed via negative feedback by supraphysiologic levels of estradiol and progesterone after ovarian stimulation, leading to severe luteal phase insufficiency [13,5].

The first studies using GnRH-agonist for final oocyte maturation reported a very poor reproductive outcome due to the severe luteolysis and it was assumed that the extend of the induced luteolysis cannot be counterbalanced by using standard luteal phase support with “only” progesterone administration [14]. However, lately several case reports demonstrate that luteolysis after GnRH-agonist trigger is not complete and varies, indicating distinct differences among patients [15–19].

This points to the fact that a new concept for individualization of luteal phase support according to the patient´s specific luteolysis might be required and could be beneficial to prevent over-treatment with hCG as well as a lack of hCG-supplementation.

In this proof of concept study, we analyzed the extend of “luteal costing” after final oocyte maturation with GnRH-agonist by measuring the progesterone-levels 84 hours / 48 hours after the application of GnRH-agonist / oocyte retrieval procedure respectively and analyzed the stimulation parameters in order to search for predictive parameters towards the prediction of luteal phase insufficiency.

## Material and methods

### Patients

In this observational, proof of concept study, we analyzed 51 patients, who were treated between August 2015 and August 2016 for primary / secondary infertility and indication for ovarian stimulation for IVF.

### Stimulation protocol

Hormonal stimulation was performed in GnRH-antagonist protocols with recombinant FSH (Puregon, MSD; Gonal F, MerckSerono) or HMG (Human Menopausal Gonadotropin) (Menogon or Menopur, Ferring). The starting dosage was chosen according to the results of the Anti-Mullerian-Hormone (AMH) as well as the antral follicle count (AFC) [20]. Starting on day 5, patients received a daily dosage of 0.25 mg GnRH-antagonist (Orgalutran, MSD or Cetrotide, MERCK) to prevent early ovulation.

During the stimulation course, stimulation dosage was adapted to the individual patient`s response. GnRH-agonist trigger for final oocyte maturation was used to avoid ovarian hyperstimulation syndrome as the ultrasound showed > 13 follicles with a size of ≥ 11 mm, which was described previously as a risk factor for OHSS-development [21]. Patients received 0.3 mg of GnRH-agonist (Decapeptyl, Ferring) for final oocyte maturation, when > 3 follicles were ≥ 17mm in diameter. OPU was performed 36 h later under general anesthesia, aspirating all follicles of a size of ≥11 mm.

### Luteal phase support

Patients started luteal phase support, using vaginal progesterone-suppositories on the evening of the OPU-day with 400 mg of progesterone. From day OPU + 1 the dosage was increased to 3 x 400 mg.

### Intervention

Blood test for hormonal assessment of progesterone (P4) 48 hours after OPU-procedure / 84 hours after final oocyte maturation was conducted, to evaluate the severity of individual luteolysis. Measurement of the progesterone level at 48 hours after OPU-procedure in patients, who have had GnRH-agonist administration for final oocyte maturation, were run as a clinical routine to evaluate the need of additional hCG administration for luteal phase support.

Hormone levels (P4) were measured with the Cobas® 6000 analyzer system, Roche. The upper measurable limit of progesterone levels is 60 ng/ml, therefore levels above this limit will be also expressed as 60 ng/ml.

The study was approved by the Ethic Committee of IVI Middle East Fertility Clinic, Abu Dhabi, UAE (Research Ethics Committee IVI-MEREF001/2016). Due to the fact, that the herein analyzed measurements of progesterone levels were run as a clinical routine during IVF-treatment, the ethic committee waived the need for obtaining oral or written approval from each patient.

### Data analysis

Data-analysis was performed for the parameters age, total stimulation dosage of recombinant FSH and HMG as well as total dosage of GnRH-antagonist for ovarian suppression, number of stimulation days and days of suppression, levels of progesterone as well as the number of follicles with a size of ≥ 11 mm on the day of final oocyte maturation, numbers of retrieved oocytes as well as numbers of metaphase II and GV oocytes and finally the levels of progesterone 48 hours after oocyte retrieval procedure. Progesterone levels on the day of final oocyte maturation and 48 hours after OPU were available from all patients.

As HMG contains hCG, which has some luteotrophic activity and could therefore sustain the function of the corpora lutea even after GnRH-agonist trigger, additional a sub-analysis of the patients receiving FSH only, HMG only or in combination with FSH for stimulation was conducted.

Exploratory data analysis allowed evaluation data quality and detecting/correcting anomalies. Continuous variables were summarized and presented with the mean and standard deviation.

A correlation analysis and dot plots were performed to visually detect correlations between all the risk factors or independent variables and the P4 48 hours after the oocyte pickup, while the correlation statistics was conducted by means of Spearman's rank correlation coefficient (or Spearman's rho), a nonparametric measure of rank correlation (statistical dependence between the ranking of two variables).

Data were analyzed with the Social Package for Social Sciences (SPSS) 23.0 software (Chicago, Illinois) and statistical significance was established at p<0.05.

## Results and discussion

The mean age of the patients was 31.46 years (SD ± 5.45 years) with a range of 22 to 43 years of age. Mean total stimulation dosages for FSH and HMG were 1524.62 (SD ± 351.10 IU) and 2215.91 IU (SD ± 1199.22 IU) respectively, with a dosage from minimum 900 IU FSH and 1350 IU HMG to a maximum of 2725 IU for FSH and 4875 IU for HMG. The mean total amount of administered GnRH-antagonist dosage was 1.55 mg (SD ± 0.45 mg; range 0.75mg, i.e. 3 x 0.25 mg/days to 2.75 mg, i.e. 11 x 0.25 mg/days).

Ovarian stimulation was performed in average over 10.02 days whereas the mean number of days for suppression medication was 6.22 days (SD ± 1.52 days and ± 1,81 days respectively; range 7 to 14 and 3 to 11 days respectively).

Progesterone levels on the day of final oocyte maturation were 0.76 ng/ml (SD ± 0.27 mg), ranging from 0.24 ng/ml (minimum) to a maximum level of 1.50 ng/ml.

The ultrasound on the day of final oocyte maturation showed a mean number of 20.14 follicles (SD ± 5.69) (range 13 to 38 follicles). The mean numbers of retrieved oocytes were 18.92 (SD ± 6.14 oocytes) ranging from 9 to 38. Out of the retrieved oocytes, the numbers of MII, MI and GV oocytes were in median 14.88 / 0.31 and 1.76 (SD ± 5.28, 0.65 and 2.39 respectively) with a range of 4 to 29 / 0 to 3 and 0 to 13, respectively.

**Fig 1:**
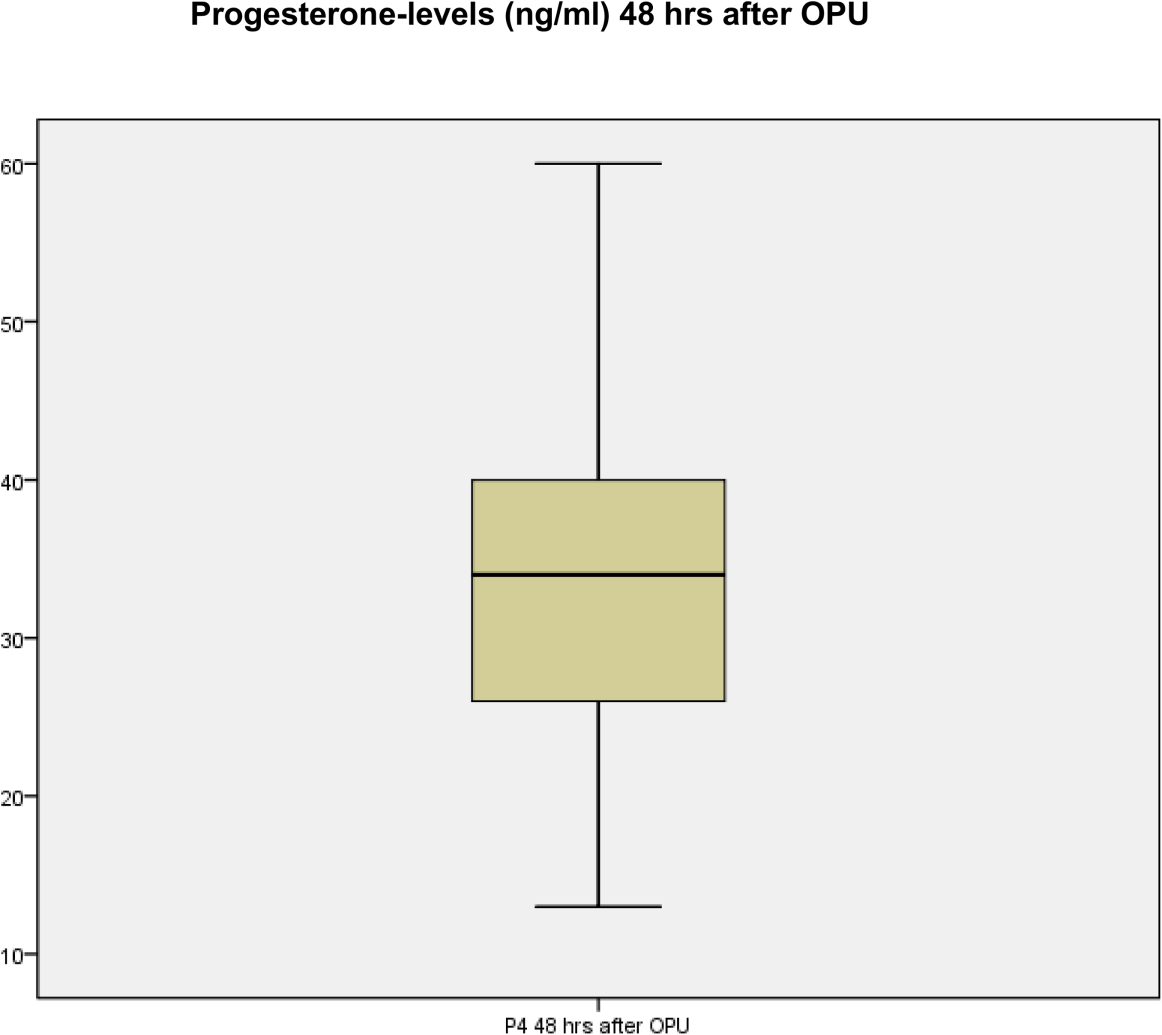
Distribution of progesterone levels 48 hours after oocyte-retrieval-procedure.

Measurement of progesterone levels 48 hours after oocyte retrieval procedure showed a mean progesterone level of 33.43 ng/ml (SD ± 10.87 ng/ml) with a range from 13.00 ng/ml to 60 ng/ml. As already mentioned, the upper measurable limit for progesterone of the analyzer was ≥ 60 ng/ml for progesterone. Table 1 shows the summary of the patient data and Fig 1 demonstrates the distribution of progesterone levels 48 hours after oocyte-retrieval-procedure.

**Table 1:** Summary of the patient-data.

No.	Age	Total stim. Dosage FSH (IU)	Total stim. Dosage hMG (IU)	Total stim. Dosage (IU)	No. Days of stim.	Total suppress-ion dose (mg)	No. Days of suppress-ion	P4-level on day of trigger (ng/ml)	No. of follicles ≥ 11 mm on the day of trigger	No. Of retrieved oocytes	No. Of oo-cytes MII	No. Of oocytes GV	P4-level 48 hrs after OPU (ng/ml)
1	36	1600		1600	11	1,75	7	0,8	22	17	16	0	40
2	25	650		650	10	1	4	0,3	14	21	18	0	27,7
3	30	1200		1200	10	1,25	5	0,9	16	20	17	0	30
4	28	1175	225	1400	9	1,25	5	0,6	30	30	26	0	18,5
5	28	2137,5		2137,5	11	1,5	6	0,8	15	14	11	0	38,92
6	27	1800		1800	11	1,75	7	0,7	16	18	14	2	43
7	40		1650	1650	8	1	4	0,5	29	10	7	1	16,2
8	29		3600	3600	12	1,75	7	0,8	21	12	7	1	40,45
9	36	900		900	8	1	4	0,79	24	19	16	1	21,8
10	36	1525		1525	10	1,5	6	1,38	24	31	19	7	38,73
11	32	350		350	9	1,25	5	0,8	22	16	12	2	51,27
12	28	1425		1425	8	2	8	0,6	18	20	16	4	43,69
13	41	1475		1475	10	1,25	5	0,9	30	12	9	3	28,9
14	28	1175		1175	8	1	4	0,66	29	27	12	13	17,95
15	31	650		650	11	1,75	7	0,75	16	18	15	0	33
16	29	1150		1150	9	1,25	5	0,76	17	9	4	1	36,1
17	30	375		375	10	1,5	6	0,46	19	18	10	6	29
18	32	1375		1375	11	1,75	7	0,61	17	16	13	0	23
19	29	1912		1912	9	1,25	5	0,9	15	13	11	1	13
20	22	1275		1275	10	1,75	7	0,8	25	27	23	1	30
21	32	1425		1425	9	1,25	5	0,8	13	13	12	1	25
22	22	1050		1050	7	0,75	3	0,78	15	16	14	1	22,2
23	28	1487		1487	8	1	4	0,68	23	20	14	6	29
24	42	150	1350	1500	14	2,5	10	0,4	15	11	9	0	38
25	29	450		450	11	2	8	0,79	17	18	16	1	40
26	27	1700		1700	9	1,25	5	0,96	16	9	7	1	29
27	32	2725		2725	11	1,75	7	0,74	19	17	12	1	40,3
28	31	1550		1550	9	1,25	5	0,28	14	15	15	0	24,8
29	41		2400	2400	12	2	8	0,91	29	38	29	4	60
30	32	400		400	10	1,75	7	0,49	22	13	13	0	31
31	33	1625		1625	12	1,75	7	0,62	27	24	21	1	44,6
32	25	1200		1200	8	1,25	5	0,67	13	20	18	0	14
33	30	675		675	12	2	8	0,9	24	29	25	0	43
34	39	1200		1200	10	1,5	6	0,5	33	23	19	1	36,4
35	34	1850		1850	12	2	8	0,63	17	21	17	2	35
36	28	1575		1575	9	1,25	5	0,95	16	24	21	2	40,5
37	23	1425		1425	10	1,5	6	0,78	19	22	16	5	39
38	37	1275		1275	11	1,75	7	1,16	23	23	18	0	60
39	41		4875	4875	14	2,5	10	0,84	15	19	17	2	32
40	43		2400	2400	12	2	8	0,25	13	11	9	2	28
41	35		1950	1950	9	1,25	5	1,17	14	22	15	1	56
42	24	2200		2200	11	1,75	7	0,76	16	13	13	0	22
43	32		2100	2100	9	1,25	5	0,24	17	14	10	1	40
44	32	1050		1050	11	2,75	11	1,5	25	27	22	4	37
45	32	1200		1200	9	1,25	5	0,83	34	25	21	3	29,5
46	26	1412		1412	10	2,75	11	0,89	22	23	20	2	47
47	28	1450		1450	10	1,5	6	0,28	14	14	7	0	22
48	24	1325		1325	9	1,25	5	1,2	22	19	14	2	38,5
49	36		2025	2025	9	1,25	5	0,82	17	15	10	0	34,5
50	38		1800	1800	9	1,25	5	1,14	27	21	12	4	34,4
51	27	1775		1775	10	1,5	6	0,56	17	18	17	0	23,5

No statistically significant correlation was found between the parameters age (p = 0.113), total stimulation dosage of recombinant FSH (p = 0.650), HMG (p = 0.479), total dosage of stimulation for combined FSH and HMG-stimulation (p = 0.171), number of follicles with a size of ≥ 11 mm on the day of final oocyte maturation (p = 0.48) and the levels of progesterone 48 hours after oocyte retrieval procedure.

However, significant positive correlations between the progesterone levels after 48 hours and the number of stimulation days (p = 0.006), the number of days of ovarian suppression (p = 0.002), the total amount of medication used for ovarian suppression (p = 0.015), the level of progesterone on the day of final oocyte maturation (p = 0.011) and the number of retrieved oocytes (p = 0.019) were present.

**Fig 2:**
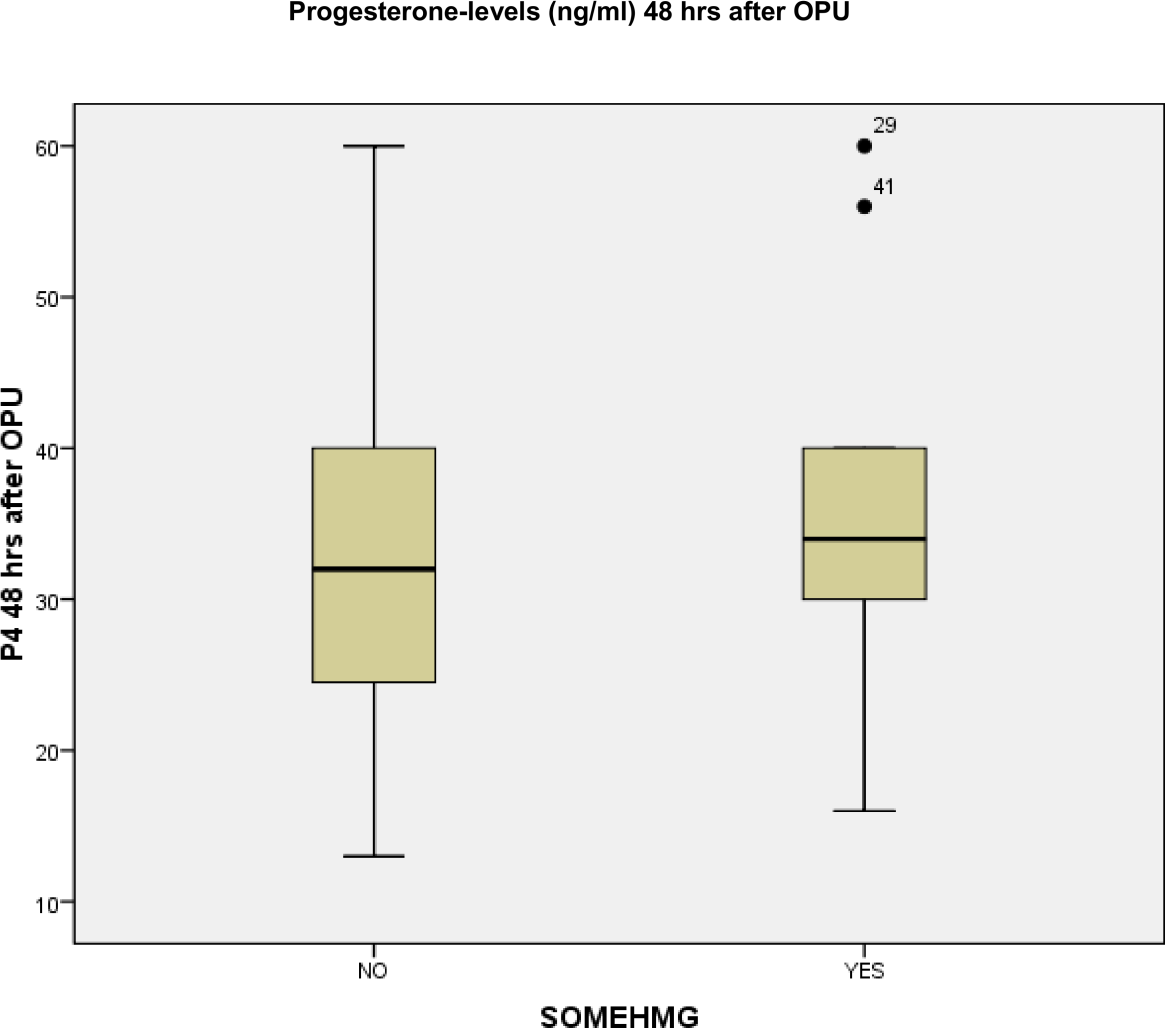
Distribution of progesterone levels 48 hours after oocyte-retrieval-procedure, depending on stimulation with or without HMG.

In the subanalysis regarding patients, who were stimulated with FSH only, HMG only or in combination with FSH, 41 patients received only recFSH, 11 patients were stimulated with HMG as sole medication and 2 patients received a combination of recFSH and HMG. The progesterone levels of the patients having recFSH and HMG as a combination for stimulation were analyzed in the HMG-group. Mean progesterone level 48 hours after OPU in the group of FSH-stimulated patients was 32.73 ng/ml (SD ± 10.16 ng/ml) and in the HMG-stimulated patients 36.00 ng/ml (SD ± 13.50 ng/ml) with a range of 13.0 to 60.0 ng/ml (FSH-group) and 16.2 ng/ml to 60 ng/ml (HMG-group) respectively. A trend towards higher progesterone levels 48 hours after OPU was seen in the HMG-stimulated patients, however, this was not statistically significant (p = 0.54). Fig 2 shows the distribution of progesterone levels 48 hours after oocyte-retrieval-procedure, depending on stimulation with or without HMG.

The analysis of progesterone-levels 48 hours post OPU-procedure / 84 hours after final oocyte maturation with GnRH-agonist of 51 patients, who underwent ovarian stimulation for IVF/ICSI-procedure in a GnRH-antagonist protocol, clearly demonstrates that luteolysis after GnRH-agonist trigger is patient-specific and emphasizes the necessity of individualized luteal phase support.

The correlation-analysis demonstrated significant positive correlations between the progesterone levels 48 hours after oocyte retrieval and the number of stimulation days, the number of days of ovarian suppression, the total amount of medication used for ovarian suppression, the level of progesterone on the day of final oocyte maturation and the number of retrieved oocytes. Therefore, longer stimulation duration as well as a higher level of progesterone on the day of final oocyte maturation and more retrieved oocytes will result in higher levels of progesterone 48 hours after oocyte retrieval, pointing towards a sustained secretory capability of the corpora lutea even after GnRHa-trigger.

Previously, it was demonstrated, that GnRH-agonist trigger for final oocyte maturation would lead to severe luteolysis [5] and additional the reproductive outcome of the first studies, using GnRH-agonist for final oocyte maturation with a “standard” luteal phase support were poor [14] as a result of severe luteolysis. However, recently published case reports [15,19] and our data demonstrate clearly that luteolysis after GnRH-agonist-trigger is individual-specific and will not follow the same pattern.

A study published by Liu et al [22] suggested that patients with progesterone levels of more than 30 ng/ml and estradiol-levels of more than 100 pg/ml at the day of implantation are more likely to have a viable and ongoing pregnancy compared to patients with hormone levels below those thresholds. Moreover, a defective luteal phase was defined if the serum mid-luteal progesterone levels are less than 10ng/ml [23].

Meanwhile the use of GnRH-agonist for final oocyte maturation is common in high responder patients as development of OHSS can almost completely be avoided. However there is an ongoing debate on the adequate luteal phase support in this scenario.

Luteolysis occurs when LH support from the primate corpus luteum is withdrawn for ≥ 3 days [24], hence corpus luteum function can be rescued if LH activity is reinitiated within 3 days, suggesting that corpus luteum viability can be preserved without LH support for at least 72 hours [25]. In order to sustain progesterone production from the corpora lutea and therefore rescue the luteal phase different treatment options have been described.

Following the concept that GnRHa-induced luteolysis can be reverted with the administration of hCG, low-doses of hCG can be given during luteal phase. However it was found, that the rescue of the corpora lutea for up to 3 days of gonadotropin deprivation is hCG dose dependent, i.e. ≥ 1,500 IU [26]. The approach of administration of 1.500 IU of hCG 35 hours after GnRH-agonist trigger resulted in comparable pregnancy rates in respect to hCG-trigger, but unfortunately with this dosage, OHSS in the high-responder-group occurred [27,28]. Castillo et al. [29] evaluated the reproductive outcome and the OHSS-incidence in a group of patients, receiving hCG in different dosages for LPS. They concluded, that low-dose HCG in the luteal phase after GnRH agonist triggering is effective in normalizing the reproductive outcome. Despite the low dosages of hCG, OHSS could not be completely avoided.

A proof-of-concept study evaluated a luteal phase support by application of daily low hCG-dosages (125 IU rec-hCG) from the day of OPU without the use of exogenous progesterone. Significant differences during the luteal phase were observed with progesterone levels being significantly higher in the groups receiving daily low-dose hCG supplementation [30]. Hence even with this low hCG dosage, OHSS occurred in 3% of the patients, however no hospitalization was needed.

OHSS can be almost completely avoided by the use of GnRH-agonist for luteal phase support. A recently published study [31] in high-responder patients used 200 mg of GnRH-agonist nasal spray twice daily (a total of 400 mg/d) for LPS after GnRH-agonist trigger without exogenous progesterone. Midluteal progesterone levels of around 190 nmol/L (approximately 59.7 ng/ml) were achieved in 97.8% of the patients and no patient developed OHSS.

Recently, a case report [32] was published with a successful pregnancy of a high-responder patient, being at risk for OHSS-development, without any administration of luteal phase support after GnRH agonist triggering for final oocyte maturation. However, progesterone- and estradiol-levels were monitored closely, in order to ensure that no luteal phase insufficiency would develop.

Our proof-of-concept study demonstrates clearly the individual pattern of luteolysis after final oocyte maturation with GnRH-agonist. This finding implies individualization of luteal phase support specific to the patient’s individual pattern of luteolysis. The weakness of the current observational study is the small number of patients, the use of different gonadotropins for stimulation and the upper limit of the progesterone measurement at 60 ng/ml.

## Conclusions

Luteal phase support after final oocyte maturation with GnRH-agonist in a GnRH-antagonist protocol is still under discussion and most patients are receiving similar luteal phase supports, despite the herein shown patient-specific pattern of luteolysis.

Contrary to the luteal phase, in the follicular phase we have understood the need to go towards individualization of ovarian stimulation, according to the patient´s specific characteristics. For a tailored luteal phase support, knowledge on predictive factors regarding a possible development of luteal phase insufficiency would be helpful in order to choose the adequate luteal phase support. According to the results of this post hoc analysis, patients with longer stimulation duration, higher progesterone-levels on the day of final oocyte maturation and more retrieved oocytes are having a smaller risk to develop rapid, severe luteal phase insufficiency.

For individualization of luteal phase support in GnRH-antagonist protocols with GnRH-agonist application for final oocyte maturation future randomized controlled studies should not only evaluate the dosage, but also the timing of hCG administration to rescue the corpora lutea.

## Supporting information

S1 Fig Distribution of progesterone levels 48 hours after oocyte-retrieval-procedure (OPU). (DOCX)

S2 Fig Distribution of progesterone levels 48 hours after oocyte-retrieval-procedure (OPU), depending on stimulation with or without HMG. (DOCX)

S1 Table Summary of the patient-data. (DOCX)
