# Supplementary material for: Individual luteolysis pattern after GnRH-agonist trigger for final oocyte maturation
Source: PLoS One. 2017 May 1;12(5):e0176600. doi: 10.1371/journal.pone.0176600 (PMC5411051; doi:10.1371/journal.pone.0176600)
Supplement: S1 Fig — (DOCX) [file pone.0176600.s001.docx]

**Supporting information**

**S1 Fig. This is the S1 Fig Title.** Distribution of progesterone levels 48 hours after oocyte-retrieval-procedure (OPU)
